# Myocardial Infarction in Polycythemia Vera and Dengue: A Case of Competing Risks

**DOI:** 10.7759/cureus.81061

**Published:** 2025-03-24

**Authors:** Maria Teresa Politi, Guido Vannoni, Juan Gagliardi

**Affiliations:** 1 Physiology, Universidad de Buenos Aires, Buenos Aires, ARG; 2 Cardiology, Hospital General de Agudos Dr. Cosme Argerich, Buenos Aires, ARG

**Keywords:** dengue, myocardial infarction, percutaneous coronary intervention, polycythemia vera, thrombocytopenia

## Abstract

A 71-year-old male with polycythemia vera, myocardial infarction, and acute dengue infection presented with intermittent chest pain, nausea, and fever. His medical history included hypertension, dyslipidemia, chronic kidney disease, and a prior myocardial infarction of unknown localization. Electrocardiography revealed ischemic changes and troponin T was significantly elevated (965 pg/mL). Given the regional dengue epidemic, NS1 antigen testing was performed and was strongly positive (64.93 S/Co; positive ≥1.0), confirming dengue. Laboratory findings included erythrocytosis (hematocrit 70.3%), mild thrombocytopenia (100,000/mm³), and worsening renal function (creatinine 1.6 mg/dL). The patient was diagnosed with a late-presenting myocardial infarction in the context of polycythemia vera-induced hypercoagulability and dengue-associated thrombocytopenia, raising critical management challenges regarding percutaneous coronary intervention (PCI) and antithrombotic therapy. Due to spontaneous symptom resolution and high bleeding risk, an immediate invasive strategy was deferred in favor of guideline-directed medical therapy and close monitoring. Platelets declined to 24,000/mm³, requiring conservative management of thrombocytopenia, phlebotomy, and fluid replacement. A delayed coronary angiography revealed an occluded proximal right coronary artery, but PCI was not performed due to lack of persistent symptoms. This case underscores the complex interplay between thrombosis and bleeding risks in high-risk cardiovascular patients, requiring a multidisciplinary approach to optimize outcomes.

## Introduction

Dengue cases have surged to record levels in 2024, with WHO documenting over 7.6 million cases and a rising mortality toll of more than 3,000 deaths in just the first few months of 2024. The virus is actively spreading in over 90 countries, highlighting its growing global impact [1]. In the United States, in 2024 there have been dengue outbreaks in Puerto Rico and the US Virgin Islands and local transmission of dengue in Florida, Texas, Hawaii, Arizona, and California [2].

Dengue, a mosquito-borne arboviral disease, poses a significant public health threat, particularly in tropical and subtropical urban areas [3,4]. It typically presents with fever, nausea, myalgia, and leukopenia, but severe cases can escalate to plasma leakage, shock, severe hemorrhage, and multi-organ failure, leading to life-threatening complications [3,4].

Myocardial infarction, a leading cause of cardiovascular mortality, requires timely antithrombotic therapy [5], while polycythemia vera, a chronic myeloproliferative neoplasm, increases thrombotic risk through hyperviscosity and abnormal blood cell activation [6].

Our case illustrates the challenges dengue infection presents in managing acute myocardial infarction in a patient with coexisting polycythemia vera, complicating risk assessment and treatment decisions.

## Case presentation

A 71-year-old male with arterial hypertension, dyslipidemia, mild chronic kidney disease (baseline serum creatinine: 1.2 mg/dL), and polycythemia vera presented to the ED during the summer in Buenos Aires, Argentina, with intermittent oppressive chest pain at rest for three days, gradually decreasing over the past 24 hours. He also reported nausea, mild abdominal pain, and a single fever episode (100.4°F) the day prior. His medical history included a myocardial infarction of unknown localization four years prior. His chronic medications consisted of aspirin (100 mg/day), atorvastatin (40 mg/day), bisoprolol (10 mg/day), and enalapril (20 mg/day).

**Figure 1:**
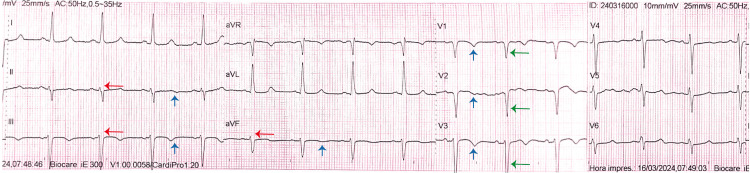
Electrocardiogram Electrocardiography showed sinus rhythm at 75 bpm, a small r wave in the inferior leads (DII, DIII, aVF) (red arrows), and a QS pattern in the anteroseptal leads (V1-V3) (green arrows). Nonspecific T-wave changes (blue arrows) were observed in the inferior (DII, DIII, aVF) and anterior (V1-V3) leads.

Vital signs were within normal limits. Physical examination revealed a plethoric face and conjunctival injection but was otherwise unremarkable. Electrocardiography (ECG) showed sinus rhythm at 75 bpm, a small r wave in the inferior leads (DII, DIII, aVF), and a QS pattern in the anteroseptal leads (V1-V3). Nonspecific T-wave changes were observed in the inferior (DII, DIII, aVF) and anterior (V1-V3) leads, suggestive of ischemia (Figure 1).

**Figure 2:**
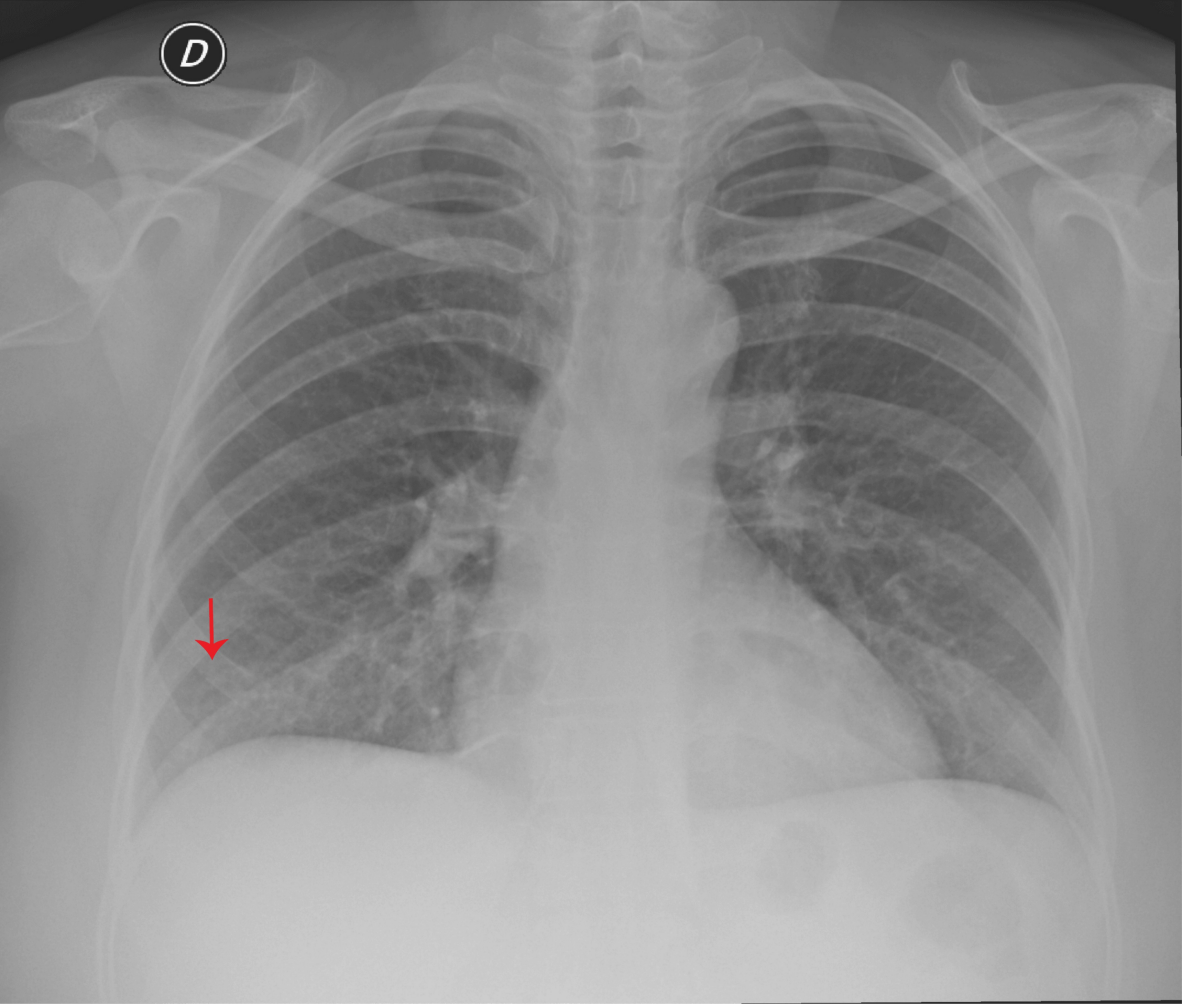
Chest X-ray The right costophrenic angle appears blunted (red arrow), indicating a possible mild pleural effusion.

Echocardiography revealed a nondilated left ventricle with concentric hypertrophy and moderately reduced systolic function, with an ejection fraction of 39% and a global longitudinal strain of -11.9%. Global hypokinesis was noted, predominantly affecting the inferolateral and septal walls. Abdominal ultrasound demonstrated mild hepatomegaly but was otherwise unremarkable. Chest X-ray showed evidence of a mild pleural effusion (Figure 2).

Polycythemia vera had been diagnosed six years earlier based on erythrocytosis (hematocrit ~70%), the presence of the JAK2 V617F mutation, and low serum erythropoietin levels (2.1 mIU/mL; normal range: 4-24 mIU/mL). The patient had discontinued hematological follow-up and treatment the previous year. His usual hematocrit was 71% (hemoglobin 23.6 g/dL) with platelets of 213,000/mm³, as recorded in a laboratory report from one year prior. On admission, his hematocrit remained similar (70%), but his platelet count had declined to 100,000/mm³, and the laboratory had technical difficulties processing the sample due to increased blood viscosity (Table 1). His chronic kidney disease had progressed with BUN 30.4 mg/dL and serum creatinine 1.6 mg/dL. Total WBC count was slightly elevated (12,080/mm³), and alanine aminotransferase was increased (99 IU/L), while aspartate aminotransferase remained normal (36 IU/L). High-sensitivity troponin T levels were significantly elevated at 965 pg/mL (normal range: <14 pg/mL) (Table 1).

**Table 1:** Laboratory results ALT: alanine aminotransferase; aPPT: activated partial thromboplastin time; AST: aspartate aminotransferase; BUN: blood urea nitrogen; INR: international normalized ratio; NS1: nonstructural protein 1; PT: prothrombin time; S/Co: signal/cutoff ratio

Lab test	Units	Reference range	One year prior	Day 1	Day 2	Day 3	Day 4	Day 5	Day 6	Day 7
Hematocrit	%	40.0-54.0	71	70.3	72.7	67.7	64.7	62.3	61.5	59.9
Hemoglobin	g/dL	13.0-18.0	23.6	23.2	25.5	23.6	22.6	22	21.5	21.3
WBC count	/mm³	4,000-11,000	10,070	12,080	12,970	13,970	13,170	10,770	10,220	10,460
Platelets	/mm³	150,000-450,000	213,000	100,000	61,000	24,000	49,000	91,000	108,000	121,000
Troponin T	ng/L	3.0-14.0	-	965.2	-	1,453.00	-	-	-	-
NS1 antigen	S/Co	<1.00	-	64.93	-	-	-	-	-	-
BUN	mg/dL	4.7-23.4	27.3	30.4	34.6	46.3	53.3	59.8	49.1	41.6
Creatinine	mg/dL	0.70-1.20	1.2	1.6	1.9	2.8	2.3	2.4	2.2	1.9
PT	s	11-13	12	35	55	47	25	16	15	15
INR	-	0.8-1.2	1	3	4.6	3.9	2.1	1.3	1.3	1.3
aPTT	s	25-39	27	52	136	64	45	33	31	32
ALT	IU/L	5-35	23	99	85	-	-	33	-	-
AST	IU/L	5-38	25	36	30	-	-	31	-	-

Given the regional dengue epidemic in South America at the time, along with the patient’s history of fever, mild thrombocytopenia, hepatomegaly, and pleural effusion, dengue was suspected. NS1 antigen (ELFA Mini Vidas method) was strongly positive (64.93 S/Co; positive ≥1.0), confirming the diagnosis (Table 1) [3,4].

## Discussion

This case was interpreted as a late presentation of an acute myocardial infarction, occurring more than 48 hours after the onset of ischemic symptoms, with elevated troponin T, ischemic changes on ECG, and imaging evidence of regional wall motion abnormality [5]. Despite the delayed presentation, due to the presence of persistent symptoms (chest pain on admission), there is consensus that prompt reperfusion therapy is indicated (Class I recommendation, Level of Evidence C) [5].

Additionally, the patient had a preexisting diagnosis of polycythemia vera, a myeloproliferative neoplasm characterized by clonal erythrocytosis, leukocytosis, thrombocytosis, and an increased risk of thrombosis [6]. The diagnosis was established based on hemoglobin levels >16.5 g/dL (men), the presence of the JAK2 V617F mutation, and subnormal serum erythropoietin levels (<4 mIU/mL) [7]. Polycythemia vera is associated with an elevated risk of both arterial (e.g., myocardial infarction) and venous thrombotic events, driven by increased hematocrit, elevated blood viscosity, abnormal platelet activation, and JAK2 mutation-mediated inflammation. Standard polycythemia vera management includes periodic phlebotomy, low-dose daily aspirin, and cytoreductive therapy with hydroxyurea for high-risk patients (age >60 years or prior thrombotic events) [5]. However, the patient had discontinued all treatments except aspirin, further increasing his thrombotic risk, thereby strengthening the indication for urgent coronary angiography and potential percutaneous coronary intervention (PCI).

Complicating the clinical picture, the patient was also in the acute phase of dengue fever. The diagnosis was suspected based on epidemiologic factors (presence in an endemic area) and clinical presentation (fever, nausea, abdominal pain, hepatomegaly, pleural effusion, and mild thrombocytopenia) and was confirmed by a positive dengue NS1 antigen test [3,4]. The severity of dengue alone warranted hospitalization, as the patient met the criteria for dengue with warning signs (abdominal pain, hepatomegaly, and pleural effusion), in addition to co-existing conditions (chronic kidney disease) and social risk (>65 years of age), which further increased his risk of complications [4].

This case presented diagnostic and management complexities due to overlapping features of myocardial infarction, dengue, and polycythemia vera. Dengue typically causes a progressive rise in hematocrit, but in this patient with preexisting erythrocytosis due to polycythemia vera, it was difficult to assess hemoconcentration as a marker of disease severity. Thus, fluid management was guided by clinical and hemodynamic parameters rather than hematocrit trends alone. Leukopenia is a hallmark of dengue infection, but in this case, the WBC count was slightly elevated, likely due to the concurrent myocardial infarction polycythemia vera-related leukocytosis, and possibly the early febrile phase of dengue. Dengue frequently leads to bleeding complications, driven by endothelial dysfunction, thrombocytopenia, coagulation abnormalities, and complement activation, resulting in increased vascular permeability, platelet dysfunction, and impaired hemostasis, often worsened by shock and organ dysfunction [3]. Although the patient had only mild thrombocytopenia on admission, platelet levels were expected to decrease further, given the temporal profile of dengue-associated thrombocytopenia [4]. Additionally, the patient had discontinued hematological follow-up and cytoreductive therapy, resulting in uncontrolled polycythemia vera, a condition associated with hyperviscosity, increased thrombotic risk, and in advanced stages, bone marrow exhaustion [6]. This latter phase may lead to cytopenias, including thrombocytopenia, which could have contributed to or amplified the platelet decline observed during the course of dengue infection in this patient. Platelet counts below 100,000/mm³ are considered a major criterion for high bleeding risk in PCI [8]. While this does not serve as an absolute contraindication to invasive reperfusion therapy, it necessitates a careful, case-by-case risk-benefit assessment [8]. Thrombocytopenia is associated with both bleeding and ischemic complications in PCI patients, adding further complexity to decision-making [9]. In addition to thrombocytopenia, dengue also induces platelet dysfunction, further predisposing to a bleeding diathesis [3]. Patients with thrombocytopenia and bleeding diathesis are often excluded from randomized trials on drug-eluting stents and dual antiplatelet therapy, making evidence-based decision-making more challenging [9]. Given these considerations, a careful reassessment of the risks and benefits of invasive reperfusion therapy was warranted.

Myocardial complications associated with dengue include both myocarditis and myocardial infarction [3]. Although the patient’s medical history included a prior myocardial infarction of an unknown location, no prior ECG or echocardiographic studies were available for comparison at the time of admission. However, in the context of acute symptoms, ischemic ECG changes, and markedly elevated troponin T, it is clinically justified to attribute the current findings, at least in part, to an acute ischemic event. While myocarditis remains a relevant differential diagnosis [10], the absence of arrhythmias, the presence of marked troponin elevation, and the patient's cardiovascular risk factors favor myocardial infarction. However, dengue-associated myocarditis cannot be entirely excluded without cardiac MRI and biopsy.

**Figure 3:**
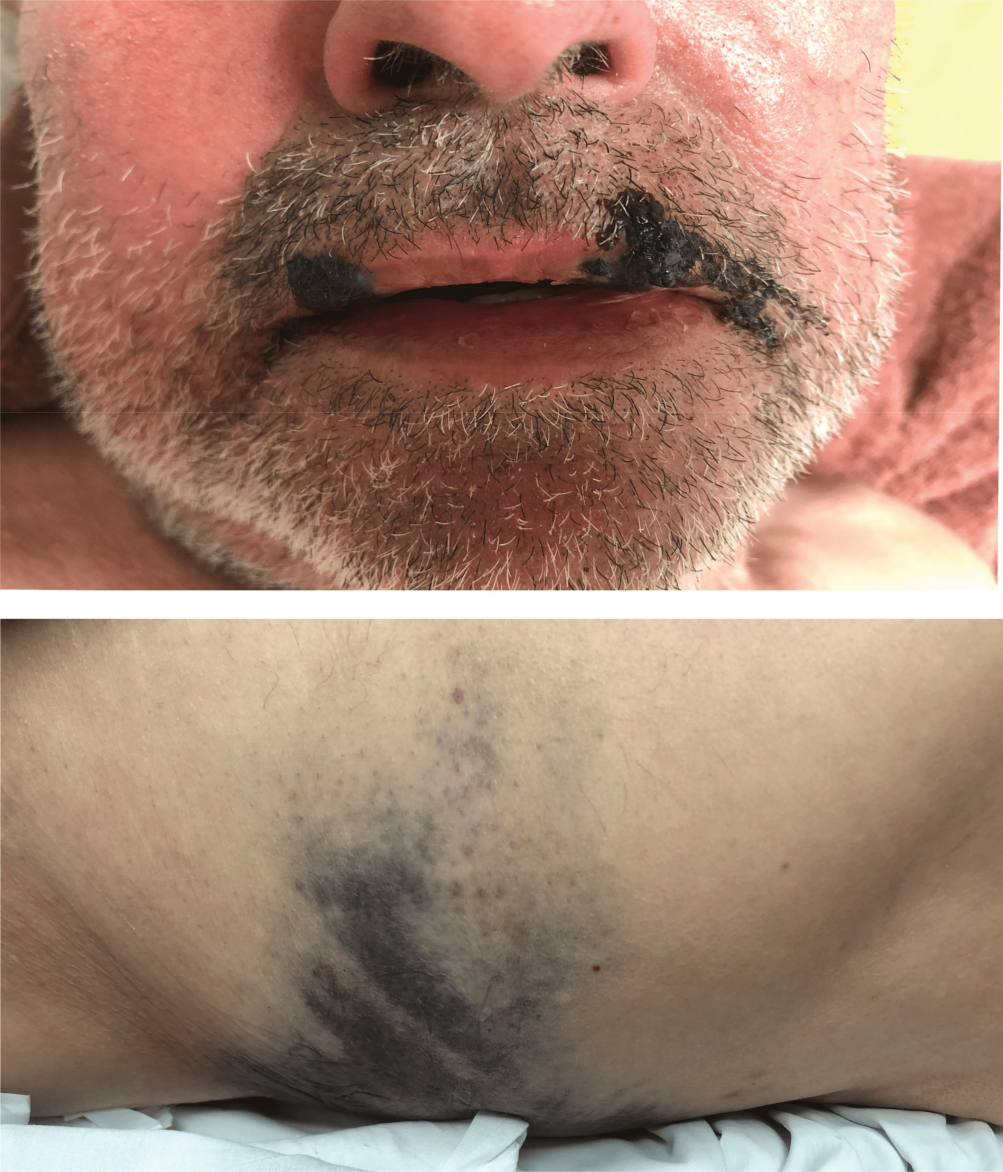
Clinical findings Oral mucosal hemorrhage (top) and ecchymosis on the lower back (bottom) were observed during hospitalization.

The patient was initiated on nitrates for symptom relief, along with guideline-directed optimal medical therapy, including dual antiplatelet therapy (aspirin and clopidogrel), anticoagulation with enoxaparin, high-intensity statins, beta-blockers, angiotensin-converting enzyme inhibitors, and aldosterone antagonists [5]. As chest pain resolved shortly after initiation of medical therapy, and considering the patient’s bleeding risk in the setting of dengue-associated thrombocytopenia, an immediate invasive reperfusion strategy was deferred in favor of close monitoring. Over the next 48 hours, coagulopathy markedly worsened, with international normalized ratio (INR) and activated partial thromboplastin time (aPTT) peaking on Day 2 (INR 4.6, aPTT 136 seconds) before gradual improvement (Table 1). Platelet counts declined progressively, reaching 61,000/mm³ on Day 2 and a nadir of 24,000/mm³ on Day 3 (Table 1). In response, clopidogrel and enoxaparin were temporarily discontinued due to the heightened bleeding risk associated with severe thrombocytopenia and coagulopathy (Table 2). The patient developed ecchymosis on his lower back and oral mucosal hemorrhage (Figure 3), which resolved with conservative management. Platelet counts gradually increased thereafter (Table 2).

**Table 2:** Clinical timeline of key events Summary of daily progression of key clinical findings, symptoms, and management decisions in a patient with concurrent dengue infection, acute myocardial infarction, and polycythemia vera. aPPT: activated partial thromboplastin time; INR: international normalized ratio; NS1: nonstructural protein 1

Day	Key symptoms/events
Day 1	Admission with chest pain, fever, nausea, mild abdominal pain; NS1 positive; mild thrombocytopenia
Day 2	Platelets decline to 61,000/mm³; INR 4.6, aPTT 136 seconds; enoxaparin and clopidogrel discontinued
Day 3	Platelet nadir 24,000/mm³; mucosal bleeding and ecchymosis develop; supportive care continued
Day 4	Renal function worsens (creatinine peaks at 2.8 mg/dL); platelets begin to recover (49,000/mm³)
Day 5	Coagulopathy improving; platelets at 91,000/mm³; patient remains stable
Day 6	Platelets rise to 108,000/mm³; renal function improving
Day 7	Continued recovery; platelets 121,000/mm³; patient hemodynamically stable

Vital signs, fluid balance, and urine output were continuously monitored to prevent volume overload or hypovolemia in the setting of dengue-associated capillary leakage. With this careful monitoring, the patient remained hemodynamically stable throughout the hospital stay. Periodic conservative phlebotomies were performed with cautious intravenous fluid replacement using lactated Ringer’s solution, taking into account the increased vascular permeability associated with dengue. These phlebotomies were performed with the primary goal of reducing hematocrit levels, thereby mitigating hyperviscosity and thrombotic risk. Despite careful fluid management, the patient developed worsening renal function peaking on Day 4 (Table 1), likely due to a combination of dengue-associated plasma leakage, systemic inflammation, and preexisting chronic kidney disease. Given the persistently elevated hematocrit despite phlebotomy, hydroxyurea was initiated to achieve better hematocrit control. Throughout hospitalization, the patient remained afebrile, asymptomatic for angina, and hemodynamically stable.

A delayed coronary angiography was performed, revealing an occluded proximal right coronary artery, confirming it as an infarct-related artery. Given the late presentation and lack of persisting symptoms, PCI was not performed. The left anterior descending and circumflex arteries exhibited only moderate mid-segment lesions, which did not warrant intervention. The patient showed clinical improvement with resolution of chest pain, stable hemodynamics, recovery of platelet counts, and no recurrent ischemic or bleeding events during hospitalization. The patient was discharged in stable condition, with recommendations for continued cardiology follow-up on optimal medical therapy and hematology follow-up with periodic phlebotomy, low-dose aspirin, and hydroxyurea.

This case underscores the challenges of myocardial infarction management in dengue-endemic regions. Our decision to defer PCI is supported by current guideline recommendations [5,8] and by stable patient progression. We believe this case can inform myocardial infarction management in regions with endemic dengue, indicating that a conservative, individualized approach can achieve a stable clinical outcome.

In similar cases, the use of systematic risk stratification tools incorporating coagulopathy markers, platelet trajectory, and ischemic risk may optimize decision-making. Furthermore, interdisciplinary collaboration is essential for tailoring strategies, particularly in dengue-endemic settings.

There is a lack of clinical trials and high-quality evidence guiding myocardial infarction management in patients with dengue-related coagulopathy, making decision-making largely expert-driven and case-dependent. Current guidelines focus on general high-bleeding-risk populations, but specific recommendations for viral coagulopathies like dengue are absent. Further prospective studies and registry data are needed to refine risk stratification, antithrombotic strategies, and PCI timing in this unique patient population.

## Conclusions

This case is unique due to the rare co-presentation of dengue infection and acute myocardial infarction in a patient with underlying polycythemia vera, a combination that creates significant diagnostic and therapeutic challenges. This case highlights the intricate clinical scenario where acute myocardial infarction coexists with polycythemia vera and dengue infection, significantly complicating therapeutic decision-making. The dual threat of thrombotic complications from polycythemia vera and the hemorrhagic risks associated with acute dengue infection posed a unique management challenge. In this context, the decision to defer immediate invasive coronary intervention and instead pursue conservative, guideline-directed medical therapy demonstrates a balanced, multidisciplinary approach tailored to minimize both bleeding and ischemic risks.

Ultimately, this case emphasizes the need for individualized care strategies in complex medical conditions, particularly when standard clinical guidelines may offer limited direct applicability. It underscores the importance of integrating hematological, infectious, and cardiovascular expertise to optimize patient outcomes, especially in the setting of overlapping and potentially competing clinical risks.

## References

[1] (2025). Dengue - global situation. Disease Outbreak News. 30 May.

[2] (2025). Current dengue outbreak. https://www.cdc.gov/dengue/outbreaks/2024/index.html.

[3] Pan American Health Organization (2025). Guidelines for the clinical diagnosis and treatment of dengue, chikungunya, and Zika. D.C.: PAHO.

[4] (2025). Dengue case management. Updated May.

[5] Byrne RA, Rossello X, Coughlan JJ (2023). 2023 ESC Guidelines for the management of acute coronary syndromes. Eur Heart J.

[6] Tefferi A, Barbui T (2023). Polycythemia vera: 2024 update on diagnosis, risk-stratification, and management. Am J Hematol.

[7] Arber DA, Orazi A, Hasserjian RP (2022). International Consensus Classification of myeloid neoplasms and acute leukemias: integrating morphologic, clinical, and genomic data. Blood.

[8] Urban P, Mehran R, Colleran R (2019). Defining high bleeding risk in patients undergoing percutaneous coronary intervention: a consensus document from the Academic Research Consortium for High Bleeding Risk. Eur Heart J.

[9] Hakim DA, Dangas GD, Caixeta A (2011). Impact of baseline thrombocytopenia on the early and late outcomes after ST-elevation myocardial infarction treated with primary angioplasty: analysis from the Harmonizing Outcomes with Revascularization and Stents in Acute Myocardial Infarction (HORIZONS-AMI) trial. Am Heart J.

[10] Cristodulo T, Gómez Martínez F, Carrillo Bayona JA, Moreno Aguilera E, Farfán Romero A Myocarditis associated with dengue virus infection: a case report. Rev Colomb Cardiol.

